# Stress Levels, Attitude toward Vaccination and Personal Protective Equipment of Students at Wroclaw Medical University during the COVID-19 Pandemic

**DOI:** 10.3390/ijerph192113860

**Published:** 2022-10-25

**Authors:** Aureliusz Andrzej Kosendiak, Bartosz Adamczak, Julia Bania, Sylwiusz Kontek

**Affiliations:** 1 Department of Physical Education and Sport, Wroclaw Medical University, Wojciecha z Brudzewa 12a, 51-601 Wroclaw, Poland; 2 Student Scientific Association at Department of Physical Education and Sport, Wroclaw Medical University, 51-601 Wroclaw, Poland

**Keywords:** stress, psychological, students, medical, pandemic, COVID-19

## Abstract

The study was conducted in March of 2021 on the students at Wroclaw Medical University from different years and faculties. Students who had mandatory physical education classes in the current year and met other eligibility criteria were enrolled. The aim of the study was to examine the impact of the pandemic on the lives of medical students. Ultimately, 660 responses to the study were included. To determine the level of stress, KPS questionnaires were used, which distinguish five types of stress. Moreover, the attitudes towards and status of vaccination were examined using PPE and declarative fettle. The findings pointed to a significantly higher level of stress in males compared to females (overall stress, 5.35 and 4.66, *p* = 0.0002), and increased external stress in some faculties (5.71 in dentistry, compared to overall 4.83, *p* = 0.009). Furthermore, medical students in their 2nd year were more stressed compared to those in their 1st year (overall stress 4.95 and 4.15, *p* < 0.0001). By knowing where the stress is highest, we can fight it more effectively and efficiently, by directing resources right where they are needed the most. A study about stress levels during the pandemic compared to physical activity should be developed.

## 1. Introduction

COVID-19 (coronavirus disease) is an infectious disease caused by the SARS-CoV-2 virus [1]. The virus is very contagious as it spreads in small liquid particles from a person’s mouth or nose when they cough, sneeze, sing, speak, or even breathe [1]. Consequently, it did not take much time to announce that coronavirus disease had transfigured into a pandemic [2]. The statement was officially published by the WHO on the 11th of March 2020 [2]. Because of the fact that COVID-19 is such an infectious disease, people were introduced to many countermeasures. It was advised to wear well-fitted masks and use an alcohol-based rub frequently [1]. Despite the fact that essential countermeasures were implemented in lots of countries, there were four waves of coronavirus disease by the end of 2021 around the world [3].

It has been proven that most Polish scholars have followed measures and countermeasures [4]. Despite the fact that lots of them have worn masks and some of them have worn gloves, they usually used disposable masks more than one time. It shows that, even though people have used personal protective equipment (PPE), they have rarely known how to do it properly [4]. Not only have people used disposable masks a couple of times, but also sometimes cloth face coverings have not been made of the right material [5]. Those actions caused the spread of the coronavirus disease. Therefore, there were four waves of growth in cases of COVID-19 in Poland within two years (2020–2022):

- October/November/December 2020
- March/April 2021
- November/December 2021
- January/February 2022 [6]

As an answer to the increasing number of coronavirus cases, on the 27th of December 2021, the Polish government enabled medical staff, employees of social welfare homes and municipal social welfare centers, and people employed at medical universities and medical students to receive the first dose of the vaccine [6]. In order to maintain social distancing, it was decided to switch to distance learning entirely and forbid carrying out lectures at universities in intramural mode [7]. The regulation was valid throughout the academic year 2020/2021. People have had different views on how the government copes with the pandemic. A survey conducted by Kantar at the beginning of October 2020 has shown that, out of all the members of the European Union, Polish citizens are some of the least satisfied with the measures their government has taken against the pandemic [8].

The situation caused by the sudden, unexpected appearance of the virus SARS-CoV-2 has drastically changed the world and people’s freedom. As research from India showed, at the beginning of the pandemic, citizens knew what the measures and PPE were. Although, people still felt that the stress was generated by reading articles about COVID-19 and constantly hearing about the virus in the media. Additionally, scientists observed obsessive thoughts about the possibility of contagion [9]. Furthermore, it has been documented that COVID-19 is explicitly associated with higher levels of both psychological and posttraumatic stress [10]. Another study pointed out the adverse influence of quarantine, self-isolation, and total lockdown on mental health. The research has reported a high prevalence of anxiety, depression, stress, PTSD, psychological disturbance, insomnia, worry, fear, OCD, and eating disorders in countries such as China, Australia, Malaysia, Iran, Germany, Spain, and Canada, all of which decided on a total lockdown [11]. Healthcare workers make up a special group that has experienced extra stressors, different than those affecting the majority of society. Serving patients with a risk of transmitting infection not only to oneself but also to members of one’s family and fear of not being resilient enough to provide the finest healthcare to many patients at once has generated additional sources of moral distress and mental burden [12]. Students belong to another special group due to their higher stress levels than the general population [13]. Their usually young age makes them more susceptible to mental health problems [14]. Scholars from countries that have not implemented significant measures against SARS-CoV-2 have a tendency to experience lower levels of stress. It might arise from a minor awareness of the pandemic. On the contrary, people studying in nations that have deployed restrictions on a huge scale are more likely to feel higher levels of stress [15]. Research shows that students have experienced stress due to uncertainty. They were not sure about the mode of study, completion of studies, or graduation and how it all would look [16]. Furthermore, the pandemic has had mostly adverse impacts on students’ financial situations, which also caused enhanced stress [16]. What is more, it has been proven that scholars who stayed active during the presence of SARS-CoV-2 had significantly lower perceived stress than inactive ones [16,17]. Additionally, less active students suffered more often from anxiety and depression than inactive ones [17].

Because of the lack of data on scholars’ well-being in Poland, this research will focus on Polish students and how they have been coping with more and more strict restrictions imposed by the Polish government over the last year. The aim of the study is also to characterize the influence of a pandemic on scholars’ stress levels.

## 2. Materials and Methods

### 2.1. Study Design and Participants

**Figure 1:**
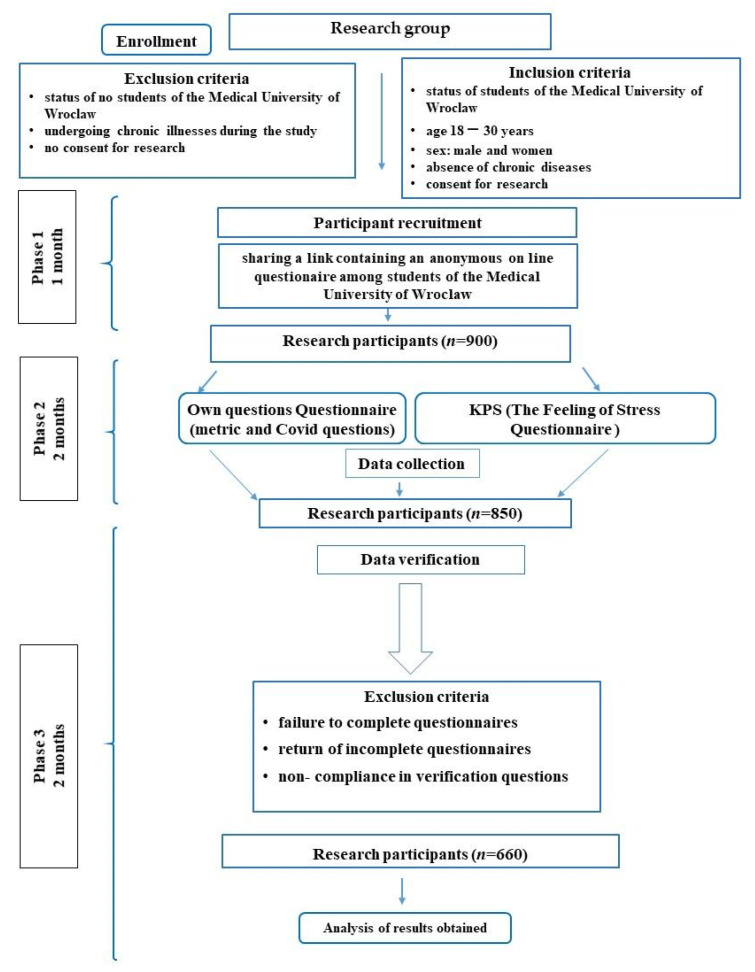
Selection process of the study.

The study was conducted in March 2021. The participant selection process is crucial for a reliably conducted study. The study size was planned to include all eligible students at Wroclaw Medical University having Physical Education lessons in the current year, in order to collect the most significant data for this cross-sectional study. The exclusion and inclusion criteria, along with the entire process of preparation and selection of participants for the study, are presented in Figure 1. In Phase 1, which lasted for 1 month, 900 people were selected as eligible for the study. Those students were notified of the invitation for the study, and the links to the online questionnaire were sent to them.

The questionnaire consisted of the Perception of Stress Questionnaire and the authors’ questionnaire, which included metric questions and questions about the COVID-19 pandemic. In Phase 2, 850 responses were collected. In Phase 3, all responses were verified. Responses in which participants failed to complete questionnaires, returned incomplete questionnaires, or where non-compliance in verification questions was identified were removed. As a result, 660 respondents were accepted after the entire verification process. Possible non-response to all questions in the questionnaire and chronic diseases in the respondents were exclusion criteria, as the results would have been insignificant for this study [18].

The difference in the number of responses obtained between the genders was expected, as the vast majority of Wroclaw Medical University students are females.

Participation in the research was voluntary, anonymous, and had the approval of the Bioethics Committee at the Wroclaw Medical University (No. KB-251/2020).

The diagram below shows the entire process of preparation and selection of study participants (Figure 1).

### 2.2. The Feeling of Stress

The Perception of Stress Questionnaire (KPS) [19] was used to determine the level of psychological stress. It contains 27 statements, to which the participants must refer with the use of a 5-step scale (1—true; 5—not true). The results are presented on five scales: Emotional Tension (E.T.), External Stress (E.S.), Internal Stress (I.S.), Lie Scale (L.S.), and Overall Stress (O.S.).

The emotional tension scale (Cronbach α = 0.82) describes experiencing the feelings of anxiety, struggling to relax, and excessive nervousness. Often it is accompanied by the abandonment of tasks and a lack of energy to act without any cause. The external stress scale (Cronbach α = 0.76) describes the feelings of frustration and tiredness resulting from the feeling that is set by the tasks of the external world (other people and society) that exceed one’s abilities. Moreover, it could be accompanied by a feeling of being used or unfairly treated. The internal stress scale (Cronbach α = 0.79) describes conflict with oneself. The feeling of worry and a lack of sense in life, caused by difficulties in overcoming the obstacles of everyday life, judging oneself as weak psychically and not talented. The overall stress scale is formed by the sum of points gained on the E.T., E.S., and I.S. scales (Cronbach α = 0.91). The lie scale (Cronbach α = 0.61) can detect people that are hiding their flaws and crediting themselves with highly socially desirable personality traits, which are rarely evinced (e.g., I have never made empty promises). This tendency could be caused by a low level of criticism.

The received raw score was turned into a sten score with the use of tables standardized for Polish society and gender.

Respondents completed an anonymous questionnaire on feelings of stress (KPS) via an online route, following all the procedures.

### 2.3. Own Questions Questionnaires

The questions in the “metrics” section included basic information about the participants: age, gender, body height, body weight, and place of residence according to city size. In addition, the body mass index was calculated. The last part of our questions concerned the COVID-19 pandemic. BMI was calculated using the data declared by individual participants. It was not measured using the bioimpedance method due to the limitations of COVID. This group of questions examined the impact of the pandemic on students in March 2021, after one year of the COVID-19 pandemic. Respondents answered questions about their status, i.e., the restrictions imposed on them, their well-being, and the real impact on themselves. The reliability of the metrics and COVID-19 of the related questions was calculated using the Cronbach-Alpha test.

### 2.4. Statistical Analysis

Excel (Microsoft, Redmond, WA, USA) was used for statistical processing and the search for significant dependencies. The sten scores obtained in the survey had a distribution that differed from the norm (an assessment of the correspondence of the obtained values to the normal distribution of the variation series using the Shapiro–Wilk W-test). A descriptive analysis was performed with frequency and percentages for the qualitative variables and with mean + standard deviation (±SD) for quantitative variables. To compare sten stress scores between two variables, the Mann–Whitney U test was used, for three or more variables, the Kruskal–Wallis test was used. For comparison between nominal variables (such as BMI and COVID-19 recovery status), the Chi-square (χ^2^) test was performed. The significance level for all the analyses was set at *p* < 0.05.

## 3. Results

A total of 660 students participated in the study, including 136 (20.6%) men and 524 (79.4%) women. Most people declared that they had inhabited a city with more than 500,000 inhabitants—203 (30.8%) and a rural area—196 (29.7%). The fewest people chose the answers “city 100,000–500,000” (55 people—8.3%) and “city below 20,000” (60 people—9.1%). For both “city 20,000–50,000” and “city 50,000–100,000”, 73 responses were obtained (11.1% each).

The body mass index value of the respondents was also examined. The vast majority, 494 people (74.8%), achieved the “normal” BMI value. However, 84 survey participants (13%) obtained a result indicating overweight; 17 people (2.6%) were struggling with obesity; 51 (7.7%) underweight participants reached a BMI score equal to 17–18.49; and 12 respondents (1.8%) were classified as underweight with a BMI score below 17.

At that time, only 115 respondents (17.4%) had been infected with COVID-19, and the remaining 545 people (82.6%) had not. There were 391 vaccinated people in the study group (59.2%). A total of 269 study participants (40.8%) had not taken any dose of the vaccine at any given time.

When asked about the impact of the pandemic on their well-being, 275 respondents (41.7%) found it difficult to say, and 316 people (47.9%) admitted that the pandemic had a negative impact on their well-being. Only 22 people (3.3%) felt the positive impact of the pandemic, and 47 people (7.1%) did not perceive any impact of the pandemic on their lives. All the above data is presented in Table 1.

Table 2 describes the fields of study of the respondents based on gender and vaccination against COVID-19. Among the survey participants, the most popular faculty was medicine (187; 28.3%), with the highest percentage of vaccinated students (157; 84%). The vast majority of people in the medical faculty were women (130; 69.5%). Nursing faculties (128; 19.4%) and pharmacy (113; 17.1%) also turned out to be large groups of respondents. Among nursing students, 72 people (56.3%) were vaccinated, and 62 were vaccinated among pharmacy students (54.9%). Most of the unvaccinated students were found in the paramedic (33.3%) and public health (17.4%) faculties.

Table 3 contains information on the correlation of the stress level on the KPS scale and the studied factors such as: gender, faculty, body mass index, recovery from COVID-19, vaccination against COVID-19, mental condition statement, and the impact of the pandemic on well-being.

When comparing the level of external stress of men (4.90), it reaches a higher value than the average value obtained for women (4.22). This result is significant (*p* = 0.0004). In the case of intrapsychic stress and the scale of lies, these values are also statistically probable (*p* < 0.00001). In both cases, men also showed an average of greater intrapsychic stress (5.52) and a higher lie scale (5.02). The general scale has a higher value for men (5.35) than for women (4.66), with *p* = 0.0002.

Moreover, the intrapsychic stress among various faculties turns out to be statistically significant (*p* = 0.009). The highest values on the KPS scale were achieved by the following fields: Dentistry (5.71), Dietetics (5.22), Public Health (5.13), and Medicine (5.06).

When comparing the BMI score with the results achieved on the KPS scale, there is a significant relationship in the scale of lies (*p* = 0.0054). The values are definitely higher, the higher the BMI score is.

Students who had been infected with COVID-19 had an average lie score (4.59) higher than those who had never developed COVID-19 infection (4.04). This difference is statistically significant (*p* = 0.0054).

In sequence, for vaccinated people, a reduced level of external stress (5.21) and intrapsychic stress (4.71) can be observed in contrast to unvaccinated people (external stress: 5.56; *p* = 0.014 and intrapsychic stress: 5.01; *p* = 0.04).

Survey participants were asked about their mental condition. Their responses have been tabulated with the average score gained on the KPS scale. Values for emotional tension, external stress, intrapsychic stress, and overall scale were statistically significant (*p* < 0.0001). People who replied that they “feel the same” reached the highest average scores in emotional tension (5.40), external stress (6.04), intrapsychic stress (5.84), and overall scale (5.84). Moreover, people who claimed that they felt happy also scored high values on average: emotional tension (5.33), external stress (5.93), intrapsychic stress (5.84), and overall scale (5.70).

Study participants were asked about the impact of the pandemic on their well-being. All the results were statistically significant (for the lie scale, *p* = 0.04, for the rest of the results, *p* < 0.0001). People who claimed that the pandemic had a positive impact on their well-being reached on average higher results for emotional tension (5.14), external stress (5.32), intrapsychic stress (5.36), lie scale (4.64), and overall scale (5.23) than participants who answered that the impact was negative. The highest scores could be observed for groups of people who marked that there was no impact on their well-being (emotional tension—5.53; external stress—6.17; intrapsychic stress—5.83; overall scale—5.94). Values for the lie scale were the lowest in all groups of survey participants.

During the study, several statements were made. There is no correlation between age and score on the KPS scale reached; also, no differences in getting over COVID-19 infection and BMI level have been found (*p* = 0.64; χ^2^ = 0.908). In addition, there is no statistically significant difference between stress level and domicile, except for external stress reached by residents of 50 000–100 000 cities (*p* = 0.045), who scored 4.95 on average on the KPS scale, compared to 5.41 in the rest.

The reasons why study participants did not get vaccinated are shown below (amount of people written in brackets):

- No vaccines/vaccination withholding in the “0” group (*n* = 33)
- Fear of vaccine side effects/complications (*n* = 13)
- Waiting for the vaccination date (*n* = 12)
- Cannot get vaccinated for health reasons—diseases that exclude vaccination (*n* = 8)
- Lack of confidence in vaccines (*n* = 5)
- Infected with COVID-19 on the scheduled vaccination date (*n* = 4)
- Do not feel the need to do so (*n* = 5)
- Did not register for the vaccination yet (*n* = 8)

Table 4 presents the number of people using various personal protective equipment and a list of people using hand sanitizers as well as protective gloves among the various fields of study. Almost every respondent (652 people; 98.9%) answered that they were washing their hands. Almost the same number of people (648; 98.2%) wore face masks outside the house. A total of 579 people (87.7%) used hand sanitizers; 99 respondents (13.3%) used gloves outside the house and only 4 (0.6%) also used them at home. The same number of people (4; 0.6%) used protective goggles. Seven of the survey participants (1.1%) wore face masks at home. Everyone claimed that they used some sort of PPE.

The faculties with the highest percentage of students using hand sanitizers were: Medical laboratory (26; 96.3%), Midwifery (25; 96.2%), Nursing (120; 93.8%), Paramedic (22; 91.7%), Dentistry (22; 91.7%), Medicine (160; 88.6%) Pharmacy (100; 88.5%), Dietetics (29; 80.6%), Physiotherapy (57; 79.2%), and Public Health (18; 78.3%).

In turn, the greatest number of students used gloves in the fields of: Medical laboratory (7; 25.9%), Public health (5; 21.7%), Paramedic (4; 16.7%), Medicine (29; 15.5%), Pharmacy (17; 15%), Nursing (16; 12.5%), Physiotherapy (6; 8.3%), Dentistry (2; 8.3%), and Dietetics (2; 5.6%). Nobody was wearing protective gloves in the midwifery direction (0).

Table 5 shows the relationship between the use of gloves as well as hand sanitizers and the stress level on the KPS score. Hand sanitizers were used by 579 people (87.7%), and gloves were used by 88 people (13.3%). Only for the lie scale, the result was statistically significant in both cases (for hand sanitizers, *p* = 0.048; for gloves, *p* = 0.0039). People who did not use personal protective equipment had a higher value on the scale of lies than those who used them. On the general scale, the result for people who used gloves was lower (4.58) than for people who did not use gloves (4.84) (*p* = 0.049). This result is also credible. The remaining statements of results are statistically insignificant (*p* ≥ 0.05).

Table 6 presents differences in values scored on the KPS scale depending on the gender of medical faculty and the academic year of medical faculty.

A total of 150 respondents had been studying medicine for 1 year, and 37 had been studying medicine for two years. The obtained results indicate a higher level of emotional tension among students in their 2nd year (1st year—4.25; 2nd year—4.86), this result is authoritative (*p* = 0.028). It is similar in the case of external stress (1 year—5.19; 2 year—6.24) and it is a statistically significant result (*p* = 0.004). The general scale is similarly significant (*p* = 0.017), on average, students in the 2nd year obtained a higher value (5.59) than students in the 1st year (4.76). The scale of lies for both age groups reached a similar level (~4.25), and this difference is not statistically significant (*p* = 0.38), as is the intrapsychic stress (*p* = 0.09), which turned out to be higher for the 2nd year (5.54) than for the 1st year (4.95).

There were 57 men and 130 women among the respondents. Men in emotional tension, intrapsychic stress, and the scale of lies achieved higher values than women (respectively, 4.79 to 4.18; 5.54 to 4.85; 5.07 to 3.90), and all these results are statistically significant (respectively, *p* = 0.024; *p* = 0.028; *p* = 0.001). External stress was also higher for men (5.51) than for women (5.35), but this result is not statistically significant (*p* = 0.34). Only in the overall scale did women have a higher mean score (5.30) than men (5.06), but it is also not a significant result (*p* = 0.05).

Table 7 shows the values obtained on the KPS scale from exercising and non-exercising people. Emotional tension for people who exercised (4.51) was higher than for those who did not exercise (3.67) (*p* < 0.0001). The situation was similar in the case of external stress, people who exercised achieved a higher average score (5.46) than those who did not exercise (4.89) (*p* = 0.003); as well as with intrapsychic stress (exercising—4.97, non-exercising—4.20) (*p* = 0.0002) and the general scale of stress (exercising—4.95, non-exercising—4.15) (*p* < 0.0001). Only in the case of the lie scale did the participants who exercised obtain a lower score (4.09) than those who did not exercise (4.34), but this result is insignificant (*p* = 0.11). The other results described in the table are statistically significant.

## 4. Discussion

This study analyzed Wroclaw Medical University students’ perceived stress due to the impact of COVID-19. A survey was conducted in March 2021. The majority of the survey’s participants were women. Most of the attendees claimed that the pandemic has had a negative influence on their well-being, and other studies have confirmed that statement. Quarantine, self-isolation, and total lockdown have generated among students a high prevalence of psychological distress: posttraumatic stress disorder and depression [20]. In addition, quarantine has redounded to the emotional detachment from their family as well as friends and has decreased their productivity and study time [20]. The research also exposed common feelings among the students. The majority of participants admitted to having a feeling of being overwhelmed. Surprisingly, a lot of people claimed that they had felt the same as before the pandemic. The survey contributed by the WHO detected other popular emotions among Polish citizens, such as: uncertainty, hope, and helplessness [21]. The survey also gave some statistics about students’ BMI. Approximately 82% of participants admitted to exercising. However, 25% of scholars had an abnormal BMI. According to other research, body mass index is not a significant and accurate predictor of excessive body fat among athletes. Therefore, the index incorrectly classified athletes with a normal percentage of fat as overweight [22]. In conclusion, some of the students whose BMI was categorized as equal or above 25 kg·m^−2^ might have had the correct weight. When it comes to vaccines against SARS-CoV-2, people who studied medicine were the most inoculated group out of all faculties at the Wroclaw Medical University. Scholars from different departments did not take that many vaccines because of the accessibility. They were interested in inoculation against SARS-CoV-2 but they could not get it due to the fact that the Medical University prioritized vaccinating students of medicine rather than scholars of any other faculty.

This research’s goal was to analyze the pandemic’s impact on different types of stress: emotional tension, external stress, intrapsychic stress, and overall stress. A lie scale was also implemented. What is more, people who had taken a vaccine had lower levels of external stress than those who had not had the chance for inoculation. KPS defines this type of stress as a “feeling of frustration, fatigue that the requirements and tasks posed by others exceed the resources, abilities, and possibilities to fulfill them”. In large part, the description matches the situation in which scholars of faculties other than medicine found themselves. They wanted to take a vaccine, but they were at the end of the queue, which started with students of medicine. That is what the inoculation procedure looked like. Furthermore, unvaccinated students had higher scores of intrapsychic stress than inoculated ones. “Thinking about the future causes anxiety, resignation tendencies, pessimism in assessing oneself and the world”—that is how KPS describes intrapsychic stress. People who were unable to vaccinate themselves might have felt anxiety about their future health state. This research also examined the difference in stress levels between people who recovered from COVID-19 and those who did not experience the ailment. In this case, the only significant result was related to the lie scale. Students who recovered from COVID-19 had a higher score on this scale than people who were not convalescent. It means that scholars who suffered from coronavirus disease were more susceptible to perceiving themselves as better, stronger beings than they actually were. They more often did not see their flaws and they more frequently took credit for presenting highly socially desirable behavior, which did not necessarily exist. Such attitudes might have arisen from the higher confidence that appeared after overcoming the coronavirus disease. Nevertheless, this is only speculation but also a charted path for further research. A similar situation applies to the correlation between BMI and the lie scale. This time, the higher the body mass index score is, the higher the lie scale score is. It might mean that people who were overweight or obese tried to assure themselves that their weight was not as big as it was. It is only an assumption that requires verification through further research. On the other hand, it is an example of psychological repression, which is a natural way of managing problems [23,24]. When it comes to the correlation between gender and stress, men had a higher level of emotional tension, intrapsychic stress, and overall stress than women. Another study confirmed this result [25]. It also added that men were more likely to negatively evaluate the campus climate, and social connectedness was more negatively related to perceived stress [25]. In addition, students who were in their 2nd academic year in the medical faculty declared higher stress levels than people who were in their 1st year. Increasing stress levels lead to professional burnout [25], which is observed among medical students [26,27,28]. Furthermore, it is proven that students’ burnout increases during the following years of study [28]. 

During the survey, participants were asked about their mental condition. In comparison to other responses, people who chose the answer: “I feel the same” were marked by the highest level of all types of stress. The answer “I feel happy” had the second highest stress score. The rest of the options, which were marked rather negatively, had lower stress scores. However, different studies showed that the loss of one’s usual routine and reduced social contacts may cause boredom, frustration, and a sense of isolation, which can be the reason for high levels of stress among people, increasing the risk of mental disorders such as thought disorders and anxiety [29,30,31,32,33,34]. Moreover, it was observed that perceived feelings of loneliness have been reported as a specific risk factor for anxiety and chronic stress [35,36,37]. By combining those facts, it can be said that anxiety and loneliness should have been associated with a higher level of stress than appeared in the relevant research. The same situation emerged when it came to the impact of the pandemic on well-being. Participants who described the influence as “positive” or “none” had the highest level of every kind of stress. This case might indicate that questions about general sensations and emotions were not specific and detailed enough so that everyone could understand them. It is advised to pinpoint questions about feelings in future research.

Participants of the survey were asked if they had used any personal protective equipment (PPE). None of them chose the answer “I do not use any”. This means that everyone applied some type of PPE to their everyday life. What is more, hand sanitizers were most common among people from faculties that had the most contact with biological materials. People who did not use hand sanitizers had a higher lie scale than those who did. This might mean that students with a high score on the lie scale did not use hand sanitizer because they did not feel the need as if they were safe without using PPE.

The last part of the survey concerned exercising. It was revealed that students who exercised had a higher level of every kind of stress than scholars who did not exercise. The result contrasts with other studies where a negative correlation between stress and sport was found [38]. There is a probability that students had done sports even before the pandemic was stated. Therefore, an announcement of total lockdown and the closure of gyms generated additional stress, which was relevant to a lack of prospects for exercising. However, it is only a conjecture which needs further verification. 

The research also had its limitations and strengths. Most of the students at the Medical University of Wroclaw are female. That is why women made up a greater part of the participants in the survey. Due to the few male attendees, answers and statistics that were assigned to male students might not have pictured their behavior, habits, and mental health well enough. When it comes to exercising praxis, it is important to understand that scholars who were in the 1st year of faculty had obligatory Physical Education classes. Those people made up the majority of the survey’s participants. This might have had an influence on answers concerning students’ exercising habits, which could be subjective and inadequate. Another limitation was the formula of the survey. Participants were answering questions online. Therefore, students might not have understood them properly, leading them to choose answers that are inconsistent with reality. Nevertheless, the research also has some strengths. It is one of the few studies that analyzes such a particular part of society as the students of medical faculties. It is important to note that the research was carried out in March of 2021, when the number of SARS-CoV-2 cases per day was among the highest, so it presents the data during one of the most serious moments. Additionally, the research presents unprecedented data on commonly asked questions.

## 5. Conclusions

This study proved that pandemic has had a negative impact on mental health on many levels. Contrary to popular opinion, it showed a significantly higher level of stress in males compared to females. Moreover, the impact of faculty has been proven—people in some faculties are more stressed than others. Interestingly, our study showed a significantly higher level of stress in people who exercised compared to those who did not. Lower vaccination rates in some of the faculties were mostly caused by difficulties in accessing vaccines. That phenomenon could have a direct impact on the increase in stress levels among students. The opposite effect might have been in getting over COVID-19, which led respondents to feel less emotional tension, external stress, and intrapsychic stress. It is highly recommended to consider the improvement of the vaccination system to avoid increasing stress levels. The pandemic affects mostly external stress (5.35 on the KPS scale), which might be correlated with the social status that is given to people associated with medicine. There are no drawbacks to the usage of PPE by medical university students; most of them at least wore face masks and washed their hands. The study also leads to further issues that need to be explored and developed in the future. Such concepts include the impact of body mass index on the scale of lies and the level of perceived stress among male medical students. Further research should also consider the expansion of knowledge about the level of stress during a pandemic in comparison with various amounts of physical activity. In addition, it is highly recommended to take care of the mental health of the community in distressing situations, such as pandemics and methods should be developed in the future study.

## Figures and Tables

**Table 1:** Characteristics of study participants.

Variables	Total, *n* = 660 (%)
*Gender*	
Male	136 (20.6)
Female	524 (79.4)
*Place of residence*	
Rural area	196 (29.7)
City < 20,000 *	60 (9.1)
City 20,000–50,000 *	73 (11.1)
City 50,000–100,000 *	73 (11.1)
City 100,000–500,000 *	55 (8.3)
City 500,000+ *	203 (30.8)
*BMI*	
Underweight (<17)	12 (1.8)
Underweight (17–18.49)	51 (7.7)
Normal (18.5–24.99)	494 (74.8)
Overweight (25–29.99)	86 (13.0)
Obesity (>30)	17 (2.6)
*Recovered from COVID-19*	
Yes	115 (17.4)
No	545 (82.6)
*Vaccinated against COVID-19*	
Yes	391 (59.2)
No	269 (40.8)
*Impact of the pandemic on well-being*	
Positive	22 (3.3)
Negative	316 (47.9)
None	47 (7.1)
Hard to decide	275 (41.7)
*Mental condition*	
I feel the same	149 (22.6)
I feel lonely	62 (9.4)
I feel anxious	31 (4.7)
I am more stressed	81 (12.3)
I cannot function	43 (6.5)
I am overwhelmed	248 (37.6)
I feel happy	46 (7.0)

Note: *n* is the number of observations; * Number of inhabitants.

**Table 2:** Characteristics of study participants based on their faculty, gender, and vaccination status.

Faculty	Total, *n* = 660 (% **)	Male, *n* = 136 (% *)	Female, *n* = 524 (% *)	Vaccinated, *n* = 391 (% *)	Unvaccinated *n* = 269 (% *)
Medical laboratory	27 (4.1)	5 (18.5)	22 (81.5)	13 (50.0)	13 (50.0)
Dietetics	36 (5.5)	2 (5.6)	34 (94.4)	11 (30.6)	25 (69.4)
Pharmacy	113 (17.1)	25 (22.1)	88 (77.9)	62 (54.9)	51 (45.1)
Physiotherapy	72 (10.9)	18 (25.0)	54 (75.0)	36 (50.0)	36 (50.0)
Medicine	187 (28.3)	57 (30.5)	130 (69.5)	157 (84.0)	30 (16.0)
Dentistry	24 (3.6)	8 (33.3)	16 (66.7)	16 (66.7)	8 (33.3)
Nursing	128 (19.4)	11 (8.6)	117 (91.4)	72 (56.3)	56 (43.8)
Midwifery	26 (3.9)	0 (0.0)	26 (100.0)	12 (46.2)	14 (53.8)
Paramedic	24 (3.6)	8 (33.3)	16 (66.7)	8 (33.3)	16 (66.7)
Public health	23 (3.5)	2 (8.7)	21 (91.3)	4 (17.4)	19 (82.6)

Note: *n* is the number of observations; * Percentage of the total number of study participants in given faculty; ** Percentage of the total number of study participants.

**Table 3:** Association between study variables and scores obtained on the KPS scale (mean score ± standard deviation).

Variables	E.T.	E.S.	I.S.	L.S.	O.S.
*Total sample*	4.36 (±1.96)	5.35 (±2.01)	4.83 (±2.14)	4.13 (±2.43)	4.80 (±1.98)
*Gender*					
Male	4.90 (±2.14)	5.63 (±1.95)	5.52 (±2.05)	5.02 (±2.42)	5.35 (±1.99)
Female	4.22 (±1.89)	5.28 (±2.02)	4.65 (±2.13)	3.90 (±2.38)	4.66 (±1.96)
*p* Value *	*p* = 0.0004	*p* = 0.058	*p* < 0.0001	*p* < 0.0001	*p* = 0.0002
*Faculty*					
Medical laboratory	4.00 (±1.33)	4.89 (±2.03)	3.78 (±1.89)	3.44 (±2.71)	4.11 (±1.67)
Dietetics	4.78 (±2.02)	5.97 (±1.78)	5.22 (±1.79)	3.69 (±2.69)	5.31 (±1.77)
Pharmacy	4.37 (±1.98)	5.40 (±2.04)	4.90 (±2.11)	4.12 (±2.49)	4.86 (±2.03)
Physiotherapy	4.26 (±2.01)	5.49 (±1.86)	4.71 (±2.25)	4.10 (±2.43)	4.83 (±1.90)
Medicine	4.37 (±2.00)	5.40 (±2.11)	5.06 (±2.14)	4.26 (±2.57)	4.93 (±2.05)
Dentistry	4.71 (±1.90)	5.04 (±2.16)	5.71 (±2.12)	4.92 (±2.39)	5.17 (±2.08)
Nursing	4.11 (±1.89)	5.10 (±1.99)	4.41 (±2.11)	4.01 (±2.15)	4.41 (±1.90)
Midwifery	4.50 (±1.63)	5.46 (±1.82)	4.69 (±1.78)	4.04 (±2.41)	4.85 (±1.62)
Paramedic	4.33 (±2.04)	5.42 (±1.84)	4.92 (±1.86)	5.08 (±2.45)	4.83 (±1.95)
Public health	5.09 (±2.54)	5.48 (±2.15)	5.13 (±2.80)	3.61 (±2.02)	5.17 (±2.61)
*p* Value **	*p* = 0.60	*p* = 0.55	*p* = 0.009	*p* = 0.15	*p* = 0.15
*BMI*					
Underweight (<17)	4.00 (±2.17)	4.50 (±2.07)	4.08 (±2.68)	3.75 (±2.26)	4.08 (±2.31)
Underweight (17–18.4)	4.59 (±1.97)	5.33 (±2.11)	5.02 (±2.70)	3.24 (±2.40)	4.94 (±1.87)
Normal (18.5–24.9)	4.35 (±1.94)	5.46 (±1.97)	4.90 (±2.13)	4.11 (±2.39)	4.87 (±1.97)
Overweight (25–29.9)	4.22 (±1.88)	5.02 (±2.08)	4.52 (±2.05)	4.71 (±2.44)	4.51 (±1.96)
Obesity (>30)	4.65 (±2.74)	4.53 (±2.07)	4.24 (±2.19)	4.88 (±2.91)	4.41 (±2.37)
*p* Value **	*p* = 0.79	*p* = 0.08	*p* = 0.22	*p* = 0.0054	*p* = 0.32
*Recovered from COVID-19*					
Yes	4.13 (±1.91)	5.15 (±2.19)	4.87 (±2.08)	4.59 (±2.29)	4.64 (±2.12)
No	4.40 (±1.97)	5.40 (±1.97)	4.82 (±2.15)	4.04 (±2.45)	4.84 (±1.95)
*p* Value *	*p* = 0.10	*p* = 0.15	*p* = 0.40	*p* = 0.0054	*p* = 0.21
*Vaccinated against COVID-19*					
Yes	4.34 (±1.99)	5.21 (±2.04)	4.71 (±2.12)	4.22 (±2.47)	4.71 (±2.00)
No	4.39 (±1.92)	5.56 (±1.95)	5.01 (±2.16)	4.01 (±2.35)	4.94 (±1.96)
*p* Value *	*p* = 0.38	*p* = 0.014	*p* = 0.04	*p* = 0.17	*p* = 0.08
*Mental condition*					
I feel the same	5.40 (±1.92)	6.04 (±1.93)	5.84 (±2.09)	3.80 (±2.17)	5.84 (±1.90)
I feel lonely	4.02 (±1.65)	4.97 (±1.96)	4.15 (±1.97)	4.32 (±2.47)	4.34 (±1.82)
I feel anxious	4.10 (±1.92)	5.45 (±1.69)	4.19 (±1.90)	4.06 (±2.41)	4.42 (±1.59)
I am more stressed	3.73 (±1.80)	5.12 (±2.01)	4.41 (±2.19)	4.37 (±2.38)	4.31 (±1.94)
I cannot function	3.26 (±1.73)	4.42 (±1.83)	3.74 (±1.88)	4.44 (±2.76)	3.63 (±1.70)
I am overwhelmed	4.06 (±1.82)	5.16 (±2.00)	4.65 (±1.97)	4.17 (±2.45)	5.45 (±1.88)
I feel happy	5.33 (±2.06)	5.93 (±2.04)	5.70 (±2.24)	4.15 (±2.78)	5.70 (±1.98)
*p* Value **	*p* < 0.0001	*p* < 0.0001	*p* < 0.0001	*p* = 0.59	*p* < 0.0001
*Impact of the pandemic on well-being*					
Positive	5.14 (±1.83)	5.32 (±2.03)	5.36 (±2.06)	4.64 (±2.68)	5.23 (±1.82)
Negative	3.80 (±1.81)	4.98 (±2.06)	4.35 (±2.11)	4.39 (±2.50)	4.29 (±1.94)
None	5.53 (±2.00)	6.17 (±2.09)	5.83 (±2.27)	3.68 (±2.35)	5.94 (±1.99)
Hard to decide	4.73 (±1.94)	5.65 (±1.85)	5.17 (±2.03)	3.88 (±2.30)	5.16 (±1.88)
*p* Value **	*p* < 0.0001	*p* < 0.0001	*p* < 0.0001	*p* = 0.04	*p* < 0.0001

Note: * value of the Mann–Whitney U test; ** value of the Kruskal–Wallis H test; E.T.—emotional tension; E.S.—external stress; I.S.—intrapsychic stress; L.S.—lie scale; O.S.—overall score.

**Table 4:** Types of PPE used by study participants and differences in using hand sanitizers and gloves between faculties.

Variable	Total, *n* = 660 (%)	
*PPE form*		
Washing hands	652 (98.9)	
Using hand sanitizers	579 (87.7)	
Using face mask outside the home	648 (98.2)	
Using gloves outside the home	99 (13.3)	
Using goggles	4 (0.6)	
Using gloves inside the home	4 (0.6)	
Using face mask inside the home	7 (1.1)	
I do not use any	0 (0.0)	
*Differences between faculties*	Hand sanitizers, *n* = 579 (% *)	Gloves, *n* = 99 (% *)
Medical laboratory	26 (96.3)	7 (25.9)
Dietetics	29 (80.6)	2 (5.6)
Pharmacy	100 (88.5)	17 (15.0)
Physiotherapy	57 (79.2)	6 (8.3)
Medicine	160 (85.6)	29 (15.5)
Dentistry	22 (91.7)	2 (8.3)
Nursing	120 (93.8)	16 (12.5)
Midwifery	25 (96.2)	0 (0.0)
Paramedic	22 (91.7)	4 (16.7)
Public health	18 (78.3)	5 (21.7)

Note: *n* is the number of observations; * Percentage of the total number of study participants in given faculty.

**Table 5:** Association between usage of hand sanitizers, gloves, and scores obtained on the KPS scale (mean score ± standard deviation).

Variables	E.T.	E.S.	I.S.	L.S.	O.S.
*Hand sanitizers*					
Using (*n* = 579)	4.33 (±1.95)	5.31 (±2.00)	4.78 (±2.11)	4.05 (±2.43)	4.76 (±1.97)
Not using (*n* = 81)	4.57 (±2.07)	5.64 (±2.08)	5.20 (±2.28)	4.75 (±2.30)	5.14 (±2.02)
*p* Value *	*p* = 0.44	*p* = 0.08	*p* = 0.12	*p* = 0.048	*p* = 0.16
*Gloves*					
Using (*n* = 88)	4.31 (±1.93)	5.05 (±1.99)	4.55 (±2.11)	3.80 (±2.47)	4.58 (±2.03)
Not using (*n* = 572)	4.36 (±1.97)	5.40 (±2.01)	4.88 (±2.14)	4.19 (±2.42)	4.84 (±1.97)
*p* Value *	*p* = 0.15	*p* = 0.054	*p* = 0.053	*p* = 0.0039	*p* = 0.049

Note: *n* is the number of observations; * value of the Mann–Whitney U test; E.T.—emotional tension; E.S.—external stress; I.S.—intrapsychic stress; L.S.—lie scale; O.S.—overall score.

**Table 6:** Differences in scores obtained on the KPS scale and year of medical faculty and gender of medical faculty.

Variables	E.T.	E.S.	I.S.	L.S.	O.S.
*Academic year of medical faculty*					
1st year (*n* = 150)	4.25 (±2.08)	5.19 (±2.13)	4.95 (±2.09)	4.26 (±2.63)	4.76 (±2.09)
2nd year (*n* = 37)	4.86 (±1.58)	6.24 (±1.80)	5.54 (±2.28)	4.24 (±2.36)	5.59 (±1.74)
*p* Value *	*p* = 0.028	*p* = 0.004	*p* = 0.09	*p* = 0.38	*p* = 0.017
*Gender on medical faculty*					
Male (*n* = 57)	4.79 (±1.93)	5.51 (±1.98)	5.54 (±2.00)	5.07 (±2.48)	5.06 (±2.26)
Female (*n* = 130)	4.18 (±2.01)	5.35 (±2.16)	4.85 (±2.17)	3.90 (±2.54)	5.30 (±1.87)
*p* Value *	*p* = 0.024	*p* = 0.34	*p* = 0.028	*p* = 0.001	*p* = 0.05

Note: * value of the Mann–Whitney U test; E.T.—emotional tension; E.S.—external stress; I.S.—intrapsychic stress; L.S.—lie scale; O.S.—overall score.

**Table 7:** Differences in score obtained on the KPS scale and exercising.

Variables	E.T.	E.S.	I.S.	L.S.	O.S.
*Exercise* (*n* = 119)	4.51 (±1.95)	5.46 (±2.00)	4.97 (±2.14)	4.09 (±2.44)	4.95 (±1.97)
*Do not exercise* (*n* = 541)	3.67 (±1.86)	4.89 (±1.98)	4.20 (±1.98)	4.34 (±2.34)	4.15 (±1.92)
*p* Value *	*p* < 0.0001	*p* = 0.003	*p* = 0.0002	*p* = 0.11	*p* < 0.0001

Note: * value of the Mann–Whitney U test; E.T.—emotional tension; E.S.—external stress; I.S.—intrapsychic stress; L.S.—lie scale; O.S.—overall score.

## Data Availability

The datasets used and/or analyzed during the current study are available from the corresponding author upon reasonable request.
